# Comparison of 25- and 27-Gauge Pars Plana Vitrectomy in Repairing Primary Rhegmatogenous Retinal Detachment

**DOI:** 10.1155/2018/7643174

**Published:** 2018-06-25

**Authors:** Keiko Otsuka, Hisanori Imai, Ayaka Fujii, Akiko Miki, Mizuki Tagami, Atsushi Azumi, Makoto Nakamura

**Affiliations:** ^1^Division of Ophthalmology, Department of Organ Therapeutics, Kobe University Graduate School of Medicine, 7-5-2 Kusunoki-cho, Chuo-ku, Kobe 650-0017, Japan; ^2^Department of Ophthalmology, Kobe Kaisei Hospital, 3-11-15 Shinoharakitamati, Nada-ku, Kobe 657-0068, Japan

## Abstract

**Aim:**

To compare the anatomic and visual outcomes of 25-gauge (25G), and 27-gauge (27G) transconjunctival sutureless pars plana vitrectomy (TSV) for the management of primary rhegmatogeneous retinal detachment (RRD).

**Design:**

A retrospective nonrandomized clinical trial.

**Methods:**

A retrospective comparative analysis of 62 consecutive eyes from 62 patients with 6 months of follow-up was performed.

**Results:**

Thirty-two patients underwent 25G TSV, and 30 patients underwent 27G TSV for the treatment of primary RRD. There was no significant difference in baseline demographic and preoperative ocular characteristics between the two groups. The initial and final anatomical success rates were 93.8% and 100% in 25G TSV and 96.7% and 100% in 27G TSV, respectively (*p*=1 and *p*=1, resp.). Preoperative best-corrected visual acuity (BCVA) (logMAR) was 0.44 ± 0.69 and 0.38 ± 0.61 for 25G and 27G TSV, respectively (*p*=0.73). The final follow-up BCVA was 0.07 ± 0.25 and −0.02 ± 0.17 for 25G and 27G TSV, respectively (*p*=0.16). The final BCVA was significantly better than the preoperative BCVA in both groups (*p*=0.02 and *p*=0.002, resp.). Preoperative intraocular pressure (IOP) (mmHg) was 13.0 ± 3.5 in 25G TSV and 14.3 ± 2.8 in 27G TSV (*p*=0.11). IOP did not statistically significantly change in both groups during the follow-up period (*p*=0.63 and *p*=0.21, resp.).

**Conclusion:**

The 27G TSV system is safe and useful for RRD treatment as 25G TSV.

## 1. Introduction

Since the transconjunctival sutureless pars plana vitrectomy (TSV) with 25-gauge (25G) and 23-gauge instrumentation was introduced, there is an accumulating trend toward TSV as the first choice for the treatment of a variety of vitreoretinal surgical indications [1–3]. The advantages of TSV including the small incision, self-sealing, decreased surgical trauma, and less postoperative inflammation provide patients better postoperative comfort, less postoperative astigmatism, and earlier visual recovery compared to the traditional 20-gauge vitrectomy system [4–8]. In 2010, Oshima et al. first reported a preliminary study regarding the safety and practicality of the 27-gauge (27G) instrument system for the vitreoretinal surgery [9]. Although the low rigidity of 27G instruments have been reported, several reports have suggested the clinical outcomes and short-term safety profile of 27G TSV for a variety of surgical indications, including rhegmatogeneous retinal detachment (RRD) [10–14]. However, there remained big concerns whether all surgeons can manage the 27G instrument especially on peripheral vitreoretinal disorders because the low instrument rigidity of 27G sometimes cause the difficulty of the operation and may impact the results especially for inexperienced surgeons.

In this study, we performed a retrospective comparative study to examine the anatomical success rates and complications of 25G and 27G TSV in the repair of primary RRD.

## 2. Patients and Methods

We performed retrospective analyses of the medical records of 62 consecutive eyes of 62 patients with primary uncomplicated RRD treated with 25G (group A) or 27G (group B) TSV. This study is a nonrandomized, retrospective comparative clinical trial. Our study was performed after obtaining the approval of the institutional review board in Kobe University School of Medicine and Kobe Kaisei Hospital. The procedure used conformed to the Tenets of the Declaration of Helsinki. Patients were enrolled from January 2014 through June 2015. Surgeries were performed by six surgeons. Ophthalmic residents in each hospital assisted all surgeries. Three surgeons who had experienced over 1000 vitreoretinal surgeries are thought to be experienced doctors, and the other three surgeons who had experienced less than 100 vitreoretinal surgeries are thought to be inexperienced doctors. Eyes with giant retinal tears, proliferative vitreoretinopathy, atopic dermatitis, and a history of prior surgery for RRD were excluded. The patients were followed up at least for 6 months after the surgery. The following variables were analyzed: sex, age, primary surgeon, preoperative best-corrected visual acuity (BCVA), postoperative BCVA, preoperative intraocular pressure (IOP), postoperative IOP, the primary anatomical success rate, the final anatomical success rate, the number of retinal breaks, locations of retinal breaks, the number of quadrants involved, the presence or absence of the macular detachment, the number of eyes who needed the suture for sclerotomy sites, the operative time, and the lens status.

### 2.1. Statistical Methods

The chi-square test and Fisher's exact probability test for dichotomous variables and unpaired *t*-test for continuous variables were used to compare parameters listed above between the two groups. Paired *t*-test was used for comparison between pre- and postoperative VA within the same group. We used the Kruskal–Wallis *H*-test to examine the transition of IOP in each group.

For logistic multiple regression analysis, univariate analysis was performed to establish the relationships between explanatory variables and the primary anatomic success rate, using the chi-square test, Fisher's exact probability test, and unpaired *t*-test. The level of statistical significance was set at *p* < 0.20. The variables found to be significant in univariate analysis were analyzed with backward logistic multiple regression analysis, using the MedCalc (MedCalc version 12.7.5.0; MedCalc Software, Mariakerke, Belgium). Statistical significance was inferred for *p* < 0.05. The Landolt decimal VA was converted to logarithmic minimum angle of resolution (logMAR) for statistical analysis.

### 2.2. Surgical Procedures

All surgeries were performed under sub-Tenon anesthesia consisting of approximately 4 mL of 2% lidocaine. The Constellation Vision system (Alcon Laboratories, Inc., Forth Worth, TX, USA) was used for both 25G and 27G TSV with a wide-angle noncontact viewing system (Resight®; Carl Zeiss Meditec AG, Jena, Germany). Three cannulas were created with conjunctival displacement and oblique-angled sclerotomies in the inferotemporal, superotemporal, and superonasal quadrants 3.0–4.0 mm posterior to the limbus. 27G twin chandelier illumination fibers (Eckardt TwinLight Chandelier, Dorc International, Zuidland, Netherlands) or 29G twin chandelier illumination fibers (Oshima dual, Synergetics USA, Inc.) were placed at 4.0 mm behind the limbus for wide-angle intraocular illumination. Before vitrectomy, for better visualization and shaving of peripheral vitreous, cataract extraction with phacoemulsification using the same machine and intraocular lens implantation were performed for all phakic eyes. Following the core vitrectomy, triamcinolone acetonide (MaQaid, Wakamoto Pharmaceutical, Tokyo, Japan) was injected to visualize vitreous gel during midperipheral vitrectomy. Then, the peripheral vitreous gel was shaved for 360° with scleral indentation under a wide-angle noncontact viewing system. No internal drainage retinotomies was made in the majority of cases to avoid the awareness of a scotoma but made in the most dependent or anterior part of the detached retina if necessary. Subretinal fluid was evacuated from original tears or drainage retinotomy sites and followed by a complete fluid-air exchange. All retinal detachments were reattached intraoperatively. Endolaser photocoagulation was applied to completely surround all retinal breaks and drainage retinotomy sites. At the end of surgery, all eyes were flushed with 50 mL of mix of 20% SF6 gas to assure a complete exchange. Additional gas mixture was injected through the pars plana to adjust IOP if necessary. Any sclerotomy sites that were found to be leaking at the end of the surgery were sutured with 8-0 vicryl suture. Normal IOP was checked by tactile examination. Subconjunctival corticosteroids were injected, and antibiotic ointment was administered at the end of the surgical procedure. All patients were kept in a prone position for 1 to 2 weeks after surgery, at least until less than 50% gas fill.

## 3. Results

Table 1 summarizes patients' perioperative demographic data. Thirty-two eyes underwent 25G TSV (group A) and 30 eyes with 27G TSV (group B). There were 24 men in group A, and 18 men in group B (*p*=0.32). The mean ± SD age was 59 ± 13 years in group A and 55 ± 9 years in group B (*p*=0.15). Their preoperative BCVA (log MAR) was 0.44 ± 0.69 units in group A and 0.38 ± 0.61 in group B (*p*=0.73). Their postoperative BCVA at the last visit was 0.07 ± 0.25 in group A and −0.02 ± 0.17 in group B (*p*=0.16). The final BCVA was significantly better than the preoperative BCVA in both groups (*p*=0.02, *p*=0.002, resp.). The initial and final anatomical success rates were 93.8% and 100% in group A and 96.7% and 100% in group B, respectively (*p*=1 and *p*=1, resp.). The mean ± SD number of retinal beaks was 1.6 ± 1.2 in group A and 1.9 ± 1.2 in group B (*p*=0.30). Twenty-three eyes had original breaks at the superior quadrant, 8 eyes at the inferior quadrant, and 1 eye at both the quadrants in group A. On the contrary, nineteen eyes had original breaks at the superior quadrant, 9 eyes at the inferior quadrant, and 2 eyes at both the quadrants in group B. There were no statistical differences in the location and the number of the breaks between both groups (*p*=0.96). Twenty-seven eyes had RRD involving one or two quadrants, and five eyes had more extensive RRD involving three or four quadrants in group A. Twenty-seven eyes had RRD involving one or two quadrants, and three eyes had more extensive RRD involving three or four quadrants in group B. There was no statistical difference in the location and extent of RRD between both groups (*p*=0.99). The macula was attached preoperatively in 23 (71.9%) eyes in group A and 15 (50%) in group B (*p*=0.13). The number of wounds that required sutures was 9/96 in group A and 4/90 in group B (*p*=0.30). The operative time (minutes) was 103.3 ± 39.9 in group A and 98.4 ± 28.3 in group B (*p*=0.77).

**Figure 1:**
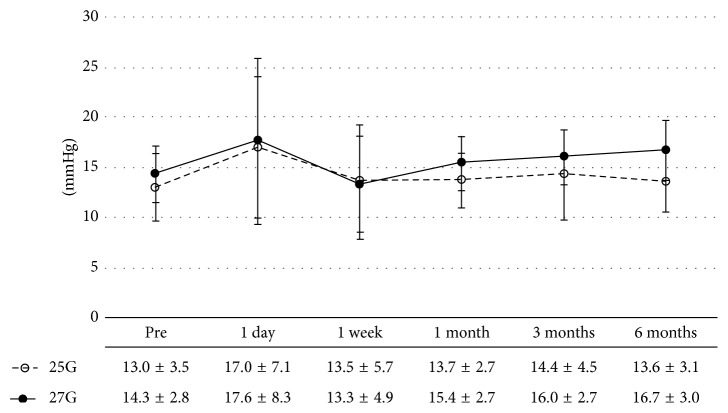
Time course changes in intraocular pressure (IOP). The mean IOP at baseline was 13.0 ± 3.5 mmHg in group A and 14.3 ± 2.8 mmHg in group B. At 1 day, 1 week, 1 month, 3 months, and 6 months postoperatively, the IOP was 17.0 ± 7.1, 13.5 ± 5.7, 13.7 ± 2.7, 14.4 ± 4.5, and 13.6 ± 3.1 mmHg, respectively, in group A, and 17.6 ± 8.3, 13.3 ± 4.9, 15.4 ± 2.7, 16.0 ± 2.7, and 16.7 ± 3.0 mmHg, respectively, in group B. IOP did not statistically significantly change in both groups during the follow-up period (*p*=0.63 and *p*=0.21, resp.).

We evaluated time course changes in IOP during the follow-up period. Preoperative IOP (mmHg) was 13.0 ± 3.5 in group A and 14.3 ± 2.8 in group B (*p*=0.11). IOP did not statistically significantly change in both groups during the follow-up period (*p*=0.63 and *p*=0.21, resp.) (Figure 1). No eye developed postoperative hypotony in both groups.

We experienced retinal redetachment in both groups (Table 2). The rate of these complications was similar between the two groups. We performed additional 27G TSV with 20% sulfur hexafluoride gas tamponade for the treatment of retinal redetachment by experienced doctors, and all eyes obtained the final retinal attachment.

As a result of univariate analysis, the following variables were selected to perform backward logistic multiple regression analysis: age, number of quadrants involved, and lens status. The logistic multiple regression analysis revealed that all variables were not associated with the primary anatomic success (Table 3).

## 4. Discussion

As previously reported [9, 15], in 27G TSV, the vitreous cutter used is short in length and low in stiffness, which bears clinicians, especially for surgically inexperienced doctors, concern regarding the possibility of compromised safety and operability in applying the 27G TSV system on the peripheral vitreoretinal diseases compared with the established larger-gauge TSV systems. In this study, we reported that the 27G TSV system provided anatomical results comparable to those obtained in 25G TSV. This result is compatible with the previous reports [13, 14]. In addition, the result of logistic regression analysis did not indicate any association between the primary anatomic success rate and explanatory variables, including the primary surgeon and gauge of vitreous cutter. These results indicate that 27G TSV have a potential for the practical use in the treatment of RRD for all surgeons, including inexperienced doctors.

Other concerns related to 27G TSV has been the low aspiration efficiency [15], which may extend the operation time. Mitsui et al. reported that, in 27G TSV, the operation time was significantly longer than that in 25G TSV for the epiretinal membrane [12]. On the contrary, previous reports suggested that the duration of vitreous removal is not different between 25G and 27G TSV for RRD [13, 14]. Our results also did not show a significant difference in operation time between 25G and 27G TSV for RRD. In terms of the core vitreoctomy process, the surgeon indeed felt that the aspiration efficiency of 27G TSV was obviously inferior to that of 25G TSV, as theoretically anticipated [15]. However, during the vitreous gel shaving in the vicinity of a detached retina, the critical procedure of the peripheral vitrectomy for RRD, 27G TSV was rather safe because of less frequent flapping of the retina due to lower aspiration efficiency compared with the 25G TSV. As a result, the total operation time did not differ between the two systems. Moreover, in the epiretinal membrane surgery reported by Mitsui et al. [12], peripheral vitrectomy was not as strenuous as that in RRD surgery. This difference in the required procedures could also account for the discrepant results obtained between the studies. Collectively, we believe that our results indicate the equivalency between 25G and 27G TSV for the RRD treatment. Accumulating cases are needed to confirm our preliminary observations.

Generally, 27G TSV requires a smaller incision, which suggests the possibility of excellent self-closing of the wound, compared to other vitrectomies with larger gauges [9, 15]. We monitored IOP for 6 months after surgery and found no significant difference during the follow-up period. We also found no significant difference in the number of wounds that required sutures between the two systems. In the 27G TSV performed by Mitsui et al. [12], as the stiffness of the cutter was low, the cutter had to be manipulated more dynamically; thus, wound closing might not have been as good as expected. For RRD, the cutter had to be manipulated more dynamically for the peripheral vitrectomy. This may be the reason why no difference was found between the two gauges with regard to the number of wounds that required sutures. In addition, as this research focused on the RRD, the surgery was completed by filling the vitreous cavity with SF6 gas in all the cases. Previous reports have indicated that when a gas tamponade was performed, the postoperative IOP was more stable than when it was not used [9, 16]. Therefore, the use of a gas tamponade may be a reason why the two gauges did not differ in IOP.

This study has potential limitations. First, it is a retrospective study, and there may have been a bias of patient selection. Another problem is that the sample size was relatively small. The results of large-scale, prospective research studies in the future are needed.

In conclusions, we performed a comparative study of outcomes between 25G and 27G TSV for RRD. The surgical results of the two gauges were equivalent with the similar effectiveness. We believe that 27G TSV is as useful as 25G for RRD, which is a disease of the peripheral retina.

## Figures and Tables

**Table 1:** Perioperative demographic data of the patients.

Characteristics	25 gauge	27 gauge	*p* value
Number of eyes	32	30	—
Sex, male/female	24/8	18/12	0.32
Age (years), mean ± SD	59 ± 13	55 ± 9	0.15
Preoperative visual acuity (logMAR), mean ± SD	0.44 ± 0.69	0.38 ± 0.61	0.73
Visual acuity at the last visit (logMAR), mean ± SD	0.07 ± 0.25	−0.02 ± 0.17	0.16
Preoperative intraocular pressure (mmHg), mean ± SD	13.0 ± 3.5	14.3 ± 2.8	0.11
Initial anatomical success	30 (93.8%)	29 (96.7%)	1
Final anatomical success	32 (100%)	30 (100%)	1
Number of breaks, mean ± SD	1.6 ± 1.2	1.9 ± 1.2	0.30
Location of breaks, superior/inferior/both/undetectable	23/8/1/0	19/9/2/0	0.96
Quadrant of retinal detachment, 1/2/3/4	10/17/4/1	10/17/3/0	0.99
Macular detachment, macular on/macular off	23/9	15/15	0.13
The number of sclerotomies that required sutures	9	4	0.30
Operative time (min), mean ± SD	103.3 ± 39.9	98.4 ± 28.3	0.77
Lens status, phakic/pseudophakic/aphakic	24/7/1	27/3/0	0.61

**Table 2:** Details of patients with postoperative complications.

Complication	25 gauge	27 gauge	Second treatment
Retinal redetachment	2	1	27GPPV + SF6 tamponade

27GPPV = 27-gauge pars plana vitrectomy; SF6 = sulfur hexafluoride.

**Table 3:** Analysis for the establishment of the relationships between explanatory variables and the primary anatomic success.

Explanatory variables	Univariate analysis	Logistic regression analysis	
	*p* value	*p* value	OR (95% CI)
Sex	0.26	NE	
Age	0.04	0.054	0.88 (0.78–1.00)
Preoperative visual acuity	0.31	NE	
Visual acuity at the last visit	0.42	NE	
Primary surgeon	0.24	NE	
Number of breaks	0.91	NE	
Location of breaks	0.44	NE	
Quadrant of retinal detachment	0.19	Not included in the model	
Macular detachment	0.28	NE	
Operative time	0.54	NE	
Lens status	0.08	Not included in the model	
Gauge of vitreous cutter	1	NE	

OR = odds ratio; CI = confidence interval; NE = not entered into multiple regression analysis.

## Data Availability

The data used to support the findings of this study are restricted by the institutional review board in Kobe University School of Medicine and Kobe Kaisei Hospital in order to protect patient privacy. Data are available from Hisanori Imai, who is the corresponding author of this manuscript, for researchers who meet the criteria for access to confidential data.
